# Degradation of Chrysene by Enriched Bacterial Consortium

**DOI:** 10.3389/fmicb.2018.01333

**Published:** 2018-06-26

**Authors:** Sagar Vaidya, Neelam Devpura, Kunal Jain, Datta Madamwar

**Affiliations:** Environmental Genomics and Proteomics Lab, UGC Centre of Advanced Study, Post Graduate Department of Biosciences, Sardar Patel University, Anand, India

**Keywords:** polycyclic aromatic hydrocarbon (PAH), bioremediation, phthalic acid, surfactants, microcosms

## Abstract

Chrysene is a high molecular weight (HMW), polycyclic aromatic hydrocarbon (PAH) known for its recalcitrance and carcinogenic properties and sparsely soluble (0.003 mg/L) in aqueous medium. Due to these refractory properties, bioavailability of chrysene is very low and therefore is persistence in the environment escaping the metabolism by microorganisms. However, few bacterial and fungal strains are reported to degrade chrysene, but with lower efficiency, requiring additional/extraneous carbon sources (co-substrates) for it’s complete mineralization. In this study, development, enrichment and characterization of bacterial consortium ASDC, consisting of *Rhodococcus* sp., ASDC1; *Bacillus* sp. ASDC2; and *Burkholderia* sp. ASDC3 were reported. Chrysene was utilized as a sole source of carbon and energy by the consortium, having maximum degradation rate of 1.5 mg/L/day and maximum growth rate of 0.125/h, under optimized conditions of pH 7.0, 37°C under aeration of 150 rpm on gyrating shaking. Chrysene degradation was unaffected in presence of other PAHs like pyrene, fluoranthene, naphthalene, phenanthrene, benzene, toluene and xylene, individually as well as in mixture. The results revealed that peptone, ammonium nitrate, sodium succinate have enhanced the chrysene degradation rate during first 24 h of experimentation, which was later on inhibited with increase in incubation time. The chrysene degradation was inhibited by mercury even at lower concentration (1 mM). The results also revealed that SDS has enhanced its degradation by 5.2-fold for initial 24 h of growth, but with increasing in the incubation period, it decreases by 1.2-fold on 7^th^ day of experimentation. The HPLC studies suggested that chrysene was degraded through phthalic acid pathway by the consortium ASDC and the stoichiometric measurements indicated the complete mineralization of chrysene. The flask scale results were validated at simulated microcosm models, where enriched consortium ASDC exhibited maximum degradation (96%) in polluted, non-sterile soil sediment, indicating that consortial strains along with indigenous metabolism showed synergistic metabolism for degradation of chrysene. Thus, the above study revealed the useful enrichment of bacterial community for synergistic degradation of PAHs (chrysene) which could be further exploited for *in situ* remediation of PAH contaminated sites.

## Introduction

Polycyclic aromatic hydrocarbons (PAHs) are ubiquitous and priority pollutants brought into the open environment predominantly due to incomplete combustion of woods, petrol, oil, and coals. Some originates from petroleum or coal deposits and volcanic events and released in environment by various anthropogenic activities like residential heating, coal gasification and liquefying plants, asphalt production and carbon black, motor vehicle exhaust, oil spillages and coke and aluminum productions, etc. (Bamforth and Singleton, 2005; Uyttebroek et al., 2007; Singh et al., 2013; Abdel-Shafy and Mansour, 2016). Based on their molecular structure, arrangement and number of benzene rings, they are classified into high molecular weight (HMW) and low molecular weight (LMW) PAHs. Since all PAHs are made up of benzene or its derivatives, they are thermodynamically very stable in the environment; which generally resists degradation by microorganisms and thereby proving toxic, mutagenic and carcinogenic to living organisms (Nayak et al., 2011).

Chrysene is a crystalline, whitish, symmetrically composed of four fused benzene rings HMW poly-aromatic hydrocarbon, produced during incomplete combustion of organic materials (like petroleum products, woods) and classified as a priority pollutant by USEPA (Hadibarata et al., 2009; Nayak et al., 2011). It is released in open environment through various anthropogenic industrial activities, oil spillages, natural forest fires, etc. and reported for being potential human toxicant and carcinogen (Hadibarata et al., 2009). Like other PAHs, chrysene undergoes volatilization, photolysis, adsorption, chemical oxidation and bioaccumulation in soils and aquifers (Hadibarata et al., 2009). However, because of the HMW of chrysene, their rate of physicochemical changes is very low contributing its persistence nature.

Among different methods available for removing PAHs from the polluted environment, bioremediation (i.e., biological) has proved to be a better alternative, because of its cost-effectiveness, ability to treat a variety of pollutants, generation of non-toxic secondary by-products and green technology. Microbial degradation (especially bacteria) is one of the major ways to remediate the environment contaminated with PAHs (Wu et al., 2016; Marchand et al., 2017). During exposure of PAHs over a long period of time, native bacterial communities evolve an enzymatic machinery to metabolize various PAHs. The rate of metabolism (degradation) may vary depending upon the presence of PAHs and type of bacterial system involved. It also gets affected by various physicochemical parameters and availability of nutrients (carbon and energy source). Therefore, prior to developing an effective bioremediation strategy, it becomes obligatory to study and optimize the effect of physicochemical parameters along with their nutritional requirements.

Moreover, it has been advocated that it is always advantageous to employ bacteria in consortium rather than in pure cultures for achieving complete mineralization of xenobiotic compounds. As with the limited genetic potential, the competence of pure cultures would not be adequate for its complete metabolism, but in form of consortium their metabolic potential enhances by many folds and mineralization of PAHs is achievable. With all advantage over other technology, bioremediation has still limited success to remove HMW PAHs such as chrysene (Marchand et al., 2017). Very few studies have reported co-metabolism of chrysene or its utilization as a sole carbon source by microorganisms (Nayak et al., 2011) and complete mineralization.

Various bacterial genera such as *Rhizomonas, Sphingomonas*, *Polyporus*, *Pseudoxanthomonas, Alkaligenes*, *Bacillus*, *Mycobacterium*, *Pichia*, *Paracoccus* are previously reported showing ability to degraded chrysene (Willison, 2004; Zhang et al., 2004; Hesham et al., 2006; Hadibarata et al., 2009; Llado et al., 2009; Dhote et al., 2010; Nayak et al., 2011; John et al., 2012). As listed in Supplementary Table S1, the degradation efficiency of each species varies with change in concentration of chrysene used, supplement of organic nutrients or surfactants, etc.

In this study, an attempt has been made to develop a bacterial consortium capable of degrading chrysene as a sole source of carbon. Different factors affecting the chrysene degradation were studied and the competence of developed consortium for PAHs degradation was assessed under soil system. Here, we propose a probable pathway consistent with complete mineralization of chrysene by characterizing the metabolites by spectrometric and chromatographic methods.

## Materials and Methods

### Development and Characterization of Consortium ASDC

The bacterial consortium ASDC was developed and enriched by culture enrichment method. Polluted soil sediments from the bank of Amlakhadi Canal, flowing across Ankleshwar Industrial Estate, Ankleshwar, were used for consortium development. Ten grams of these samples were inoculated in Bushnell Haas broth (BHB) amended with 10 mg/L of chrysene (from the stock of 1000 mg/L dissolved in acetone, 0.22 μm filter sterilized) dissolved in acetone and incubated under shaking conditions (150 rpm) at 37°C for 15 days. After 15 days of initial incubation, whole content was centrifuged at 1000 × *g* for 3 min at 4°C to discard the debris and soil particles. Fifteen to twenty milliliters of the supernatant was re-inoculated in fresh BHB amended with 10 mg/L of chrysene and incubated under same conditions for another 15 days. Sub-culturing was repeated for two more cycles to remove any debris/soil particles. After third transfer the consortial cells were harvested at 6000 × *g*, 10 min at 4°C, pellet was resuspended in minimal quantity of sterile distilled water and immediately inoculated in fresh BHB amended with 10 mg/L of chrysene and incubated under similar conditions for 15 days. Such sequential sub-culturing was repeated for more than 25 cycles without supplementing additional carbon and/or other nutrient sources. Thus, selective conditions were provided, where developed consortium can utilize chrysene as sole carbon and energy source to obtain consistent degradation and stable consortial growth.

The bacterial constituent of the consortium was identified on solid agar media. The acclimatized consortium was serially diluted and spread on Nutrient agar, Bushnell Haas agar, Bushnell Haas agar amended with 10 mg/L of chrysene and incubated for 1–8 days at different temperatures. Discrete and morphologically distinct colonies were further screened to enumerate and obtain pure bacterial cultures. Each culture was identified using 16S rRNA gene sequence, by extracting genomic DNA and amplifying the gene by eubacterial universal primers 8F and 1492R (Desai and Madamwar, 2007). The purified gene amplicons were sequenced on automated ABI 3500 Genetic Analyzer (Thermo Scientific, ABI, United States) and gene sequence was BLAST on NCBI to identify the bacterium.

### Preparation of Inoculum and Studying the Effect of Auxiliary Nutrients and Environmental Parameters on Chrysene Degradation

Consortium ASDC was grown in BHB amended with 10 mg/L of chrysene and incubated under shaking conditions (150 rpm) at 37°C till log phase. The consortial growth at log phase was harvested at 6000 × *g* for 5 min at 4°C; cell pellet was washed (once) with sterile distilled water and resuspended in minimum quantity of sterile distilled water to get an absorbance of 1.0 at 600 nm (which represents consortial cell counts to ∼1 × 10^7^ cell/ml).

Inoculum thus prepared was used for studying the effect of auxiliary nutrients and optimizing the environmental factors for chrysene degradation by consortium ASDC.

#### Effect of Auxiliary Nutrients (Co-substrates) and Intermediates

The degradation efficiency of consortium ASDC was enhanced by supplementing peptone, yeast extract, sodium succinate, ammonium nitrate (0.1%, w/v) or glucose (2.0%, w/v) in BHM. Effect of different intermediates of chrysene degradation pathway, *viz.* phthalic acid and salicylic acid (20 g/L) were also studied. Experiments were performed along with uninoculated BHM amended with 10 mg/L of chrysene and respective co-substrates as abiotic controls. All experiments were performed in triplicates.

#### Effect of Physical Factors

Physical parameters such as, temperature (30–50°C), pH (5.0–9.0), shaking speed (oxygen concentration) (50, 100, and 150 rpm) were examined for their critical effect on chrysene degradation.

#### Effect of Chemical Factors

Initial chrysene concentration (1–10 mg/L), different hydrocarbons [fluoranthene, pyrene, phenanthrene, and naphthalene (100 mg/L)], other aromatic compounds [benzene, toluene and xylene (0.1%, w/v)], heavy metals [cadmium (Cd), lead (Pb), zinc (Zn), mercury (Hg), and chromium (Cr) at 1, 5, and 10 mM concentrations], surfactants [Tween-80 and Triton X-100, Cetyl-trymethyl ammonium bromide (CTAB), sodium dodecyl sulfate (SDS)] (0.02% v/v and w/v) were studied to observe the effect of above chemical factors on chrysene degradation. During the study of effect of different hydrocarbons and other aromatic compounds, their mixture (in respective concentration, as mentioned above) were added in the medium to assess the simultaneously degradation efficiency of the consortium.

### Inoculum and Sample Preparation for HPLC

Consortium ASDC was grown in 100 ml of BHM amended with 10 mg/L of chrysene at 37°C under shaking conditions (of 150 rpm) for 7 days and 5% (v/v) of the grown consortium was used as inoculum for further degradation studies. The complete procedure for inoculum development is described in the Supplementary Information.

The degradation profile of chrysene and its degraded intermediates were analyzed using HPLC (Prominence LC system, Shimadzu, Japan). Under optimized conditions, consortium was grown in BHB amended with 10 mg/L of chrysene. At different interval of time entire content (100 ml) was mixed with 20 ml of dichloromethane and incubated under shaking conditions at 150 rpm for 60 min. Post-incubation, the aqueous phase was allowed to separate under static conditions and 500 μl of separated organic phase (i.e., dichloromethane) were collected in the fresh tube. The organic phase was evaporated under vacuum using SpeedVac (Thermo Electron Corporation, Waltham, MA, United States) and the dried pellet was resuspended in 1 ml of 70% acetonitrile and further appropriately diluted to bring the metabolite concentration in the range of 10 ± 3 mg/L (for comparing it with the standard of 10 mg/L chrysene). Remaining chrysene and its degraded products were studied by using Pursuit 3 PAH C_18_ reverse phase column (100 mm × 4.6 mm, 3 μm) (Agilent, United States), with acetonitrile:water (70:30, v/v) as eluent and isocratic flow rate of 1 ml/min under ambient conditions was maintained. Chrysene standard and degraded products were detected at 254 nm by Photo Diode Array Detector.

### Microcosm Studies

To assess the competence of consortium ASDC for degradation of chrysene in soil system, microcosm experiments were performed as described in Pathak et al. (2009, 2011) with few modifications. Consortium ASDC was developed from the polluted soil sediments of Amlakhadi canal [where sediments are constantly submerged under water], thus, to mimic the natural conditions, 50 g of polluted and non-polluted soil samples were added in 100 ml BHM and inoculated with consortial cell concentrations of 5 × 10^7^/ml from exponential phase of consortium. As described in **Table 1**, 12 different experimental sets were performed in triplicates for 7 days and samples were withdrawn on the 3^rd^ and 7^th^ day. To estimate the remaining chrysene in the soil system, 100 ml of content was extracted in 20 ml of *n*-hexane and agitated at 120 rpm for 15 min. After allowing it to settle for 15–20 min, the supernatant was transferred to fresh tubes and appropriately diluted so as to make the concentration of metabolites in the range of 10 mg/L (±2). PAH Degradation was measured spectrophotometrically using Double Beam Specord^®^ 210 BU UV-vis spectrophotometer (Analytica Jena AG, Germany) in the spectral range of 190–500 nm against blank of *n*-hexane.

**Table 1:** The effect of indigenous microflora and the ability of the consortium ASDC for chrysene degradation during microcosm studies.

Experimental sets	Experimental parameters	Degradation (%)
Set 1	Pristine, non-sterile soil amended with 100 mg/kg pyrene, 100 mg/kg fluoranthene, 500 mg/kg naphthalene, 250 mg/kg phenanthrene and 5 mg/kg chrysene and consortium ASDC	60
Set 2	Pristine, non-sterile soil amended with 5 mg/kg chrysene and consortium ASDC	85
Set 3	Pristine, non-sterile soil amended with 5 mg/kg chrysene, to determine the ability of indigenous microflora for chrysene degradation	54
Set 4	Polluted, non-sterile soil amended with 100 mg/kg pyrene, 100 mg/kg fluoranthene, 500 mg/kg naphthalene, 250 mg/kg phenanthrene and 5 mg/kg chrysene and consortium ASDC	67
Set 5	Polluted, non-sterile soil amended with 5 mg/kg chrysene, and consortium ASDC	96
Set 6	Polluted, non-sterile soil amended with 5 mg/kg chrysene, to determine the ability of indigenous microflora for chrysene degradation	73
Set 7	Pristine, sterile soil amended with 100 mg/kg pyrene, 100 mg/kg fluoranthene, 500 mg/kg naphthalene, 250 mg/kg phenanthrene and 5 mg/kg chrysene and consortium ASDC	43
Set 8	Pristine, sterile soil amended with 5 mg/kg chrysene and consortium ASDC	68
Set 9	Pristine, sterile soil amended with 5 mg/kg chrysene, to determine abiotic loss of chrysene	01
Set 10	Polluted, sterile soil amended with 100 mg/kg pyrene, 100 mg/kg fluoranthene, 500 mg/kg naphthalene, 250 mg/kg phenanthrene and 5 mg/kg chrysene and consortium ASDC	83
Set 11	Polluted, sterile soil amended with 5 mg/kg chrysene, and consortium ASDC	89
Set 12	Polluted, sterile soil amended with 5 mg/kg chrysene, to determine abiotic loss of chrysene	03

*Polluted soils samples were collected from Amlakhadi canal, flowing across Ankleshwar Industrial Estate, Ankleshwar.*

**Table 2:** The stoichiometric correlation between chrysene and its degraded products.

Days	Chrysene (μM)	Degradation (%)	Derivative of phthalic acid (μM)
0	21.5 (5 mg/L)	—	—
3	5.00 (1.22 mg/L)	76	1.00 (0.22 mg/L)
7	0.90 (0.21 mg/L)	96	1.50 (0.31 mg/L)

### Statistical Analysis

All experiments during the degradation studies were performed in triplicates. The results were described as the mean of ± standard error means (SEM). The data were analyzed by one way analysis of variance (ANOVA), followed by Tukey–Kramer multiple comparisons test. Results were considered significant at *P*≤ 0.05 of confidence. Followed by ANOVA, linear regression kinetics was used to assess the fitness for linearity between chrysene degradation and environmental parameters.

## Results and Discussion

### Enrichment and Development of Bacterial Consortium ASDC and Identification of Bacterial Cultures

Chrysene degrading bacterial consortium ASDC was developed through culture enrichment method using polluted sediments from Amlakhadi canal. Enhanced degradation of chrysene and consistent consortial growth of the consortium ASDC was observed after each successive transfer in the fresh medium. During the process of consortial development, samples were withdrawn to assess its degradation efficiency and an appropriate quantity of consortium was spread on different media to identify the constituent bacterial composition of the consortium. Colonies with distinct morphology were further purified and on the basis of 16S rRNA gene sequencing, it was determined that consortium ASDC was comprised of three bacterial cultures namely: *Rhodococcus* sp. ASDC1 (NCBI Accession No.: MG940979), *Bacillus* sp. ASDC2 (MG940980) and *Burkholderia* sp. ASDC3 (MG940981).

### Effect of Chrysene on the Growth Rate of Consortium ASDC

It is observed that in a polluted environment microorganisms react differently toward different environment pollutants and the type of response also depends on the concentration of the pollutants (Leahy and Colwell, 1990; Boopathy, 2000). Various studies suggest that there is always a threshold concentration for every xenobiotic compounds, which directly affect their degradation by microorganisms. If their concentration is below the threshold value, it cannot be sensed by microorganisms, while if the concentration is too high (i.e., above the threshold value), the pollutant will prove toxic to native microorganisms (Doick et al., 2005; Okpokwasili and Nweke, 2005). And, it is well-established that, as the concentration of the pollutants increases (but in a confined range) the rate of degradation of pollutant also increases. Hence, the kinetic parameters *viz.* specific growth rate (*μ*), specific degradation rate (*q*) and half saturation rate constant (*k*) can provide useful information to determine the concentration of PAHs that can be degraded by the developed consortium.

**FIGURE 1:**
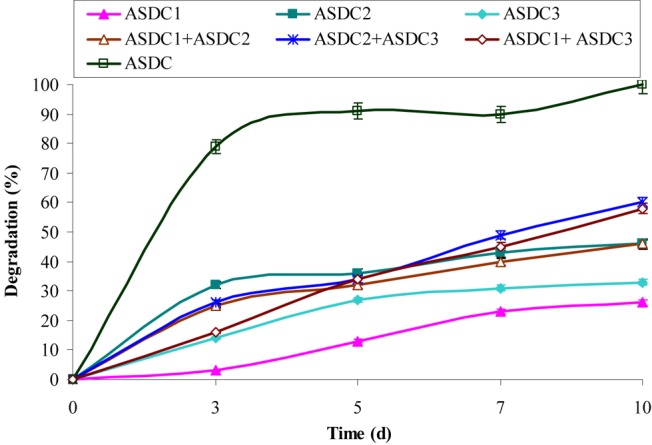
Degradation of chrysene (10 mg/L) by individual constituent pure bacterial cultures of the consortium, combination of the pure cultures and consortium under optimized growth conditions (37°C and pH 7, 150 rpm) in BHM. ASDC1: *Rhodococcus* sp. ASDC1, ASDC2: *Bacillus* sp. ASDC2, ASDC3: *Burkholderia* sp. ASDC3, ASDC: Consortium.

**FIGURE 2:**
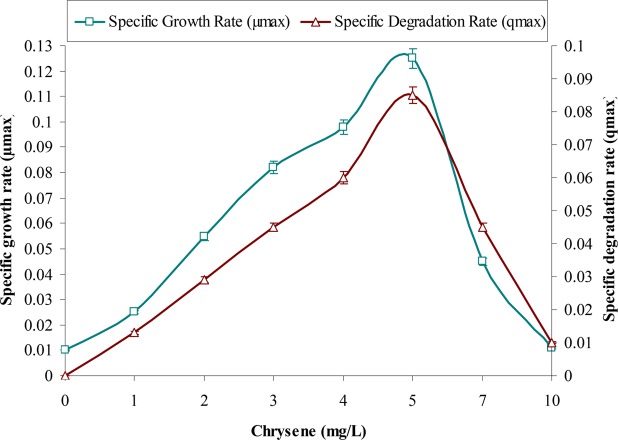
Effect of chrysene concentrations on growth rate (*μ*_max_) of the consortium ASDC and (its) degradation rate (*q_max_*), at 37°C and pH 7, 150 rpm in BHM.

This study supports the above observations and from **Figure 1** it can be observed that with increases in initial concentrations of chrysene, specific degradation rate (*q*) and specific growth rate (*μ*) of the consortium increases linearly, in the concentration range between 1 to 5 mg/L. Maximum degradation was observed at 5 mg/L. But, both these rates decreased sharply at a higher concentration from 5 to 10 mg/L, where chrysene degradation drops by nearly 1.9 (from 0.085 to 0.045 mg/L/d) and 8.5-fold (0.01 mg/L/d) for 7 and 10 mg/L of chrysene respectively. Maximum degradation rate (*q*_max_), maximum growth rate (*μ*_max_) and half saturation coefficient (*K*_S_) of chrysene for consortium ASDC were 1.5 mg/L/day, 0.125/h and 0.1 mg/L respectively. Along with the decrease in the degradation rate, growth rate also decreased with increase in chrysene degradation (**Figure 2**). The maximum growth rate was observed at 5 mg/L, while it decreases sharply by 2.8-fold at 7 mg/L (from 0.125 to 0.045/h), 11.5-fold at 10 mg/L.

Therefore, the observed results suggested that metabolism of chrysene was dependent on the growth of the consortium and is biologically mediated rather than an abiotic loss. The study further revealed that higher concentrations of chrysene were inhibitory, retarding the growth of the consortial bacteria. This experiment provided the significant observations about the substrate sensitivity of the consortium ASDC, thus the further study was performed at 5 mg/L of initial chrysene concentration.

### Effect of Organic Carbon Sources, Intermediates, and Surfactants on Chrysene Degradation

**FIGURE 3:**
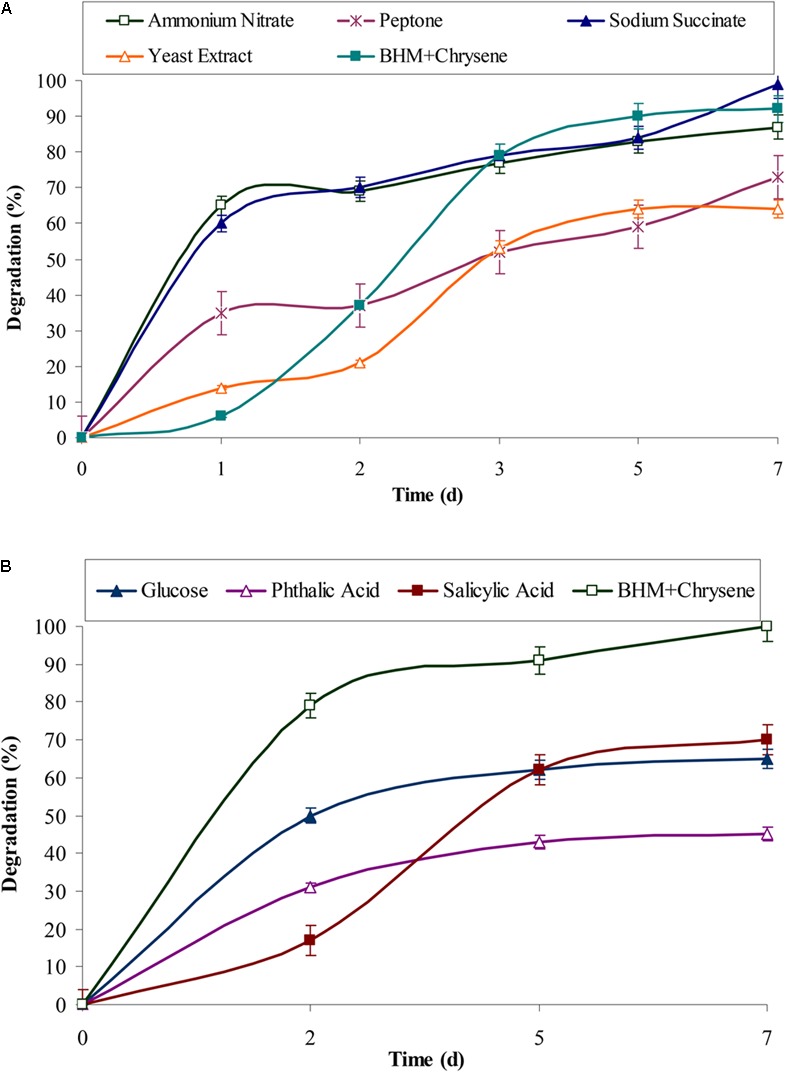
Effect of **(A)** various organic and inorganic sources and **(B)** glucose and intermediates (of PAHs degradation) on chrysene degradation by the consortium ASDC under optimized conditions (37°C and pH 7, 150 rpm) in BHM.

Polycyclic aromatic hydrocarbons usually require electron rich reduced compounds which can catabolically provide free electrons during its degradation. The observed results from **Figures 3A,B** showed that rate of degradation of chrysene was mostly unaffected in presence of succinate (*P* < 0.05), while nitrate had a negligible effect on chrysene degradation. Peptone, yeast extract, and glucose had an inhibitory effect on its degradation, the rate of degradation decreased by 1.27, 1.14, and 1.55 (*P* < 0.05) fold respectively. On providing intermediatory compounds of PAHs degradation pathway, i.e., phthalic acid and salicylic acid, chrysene degradation was again decreased by 2.2- and 1.4-fold respectively. In our earlier study on pyrene degradation by the bacterial consortium ASDP, similar results were observed (Vaidya et al., 2017). The decrease in degradation rate could be attributed to feedback inhibition of enzymes involved in the metabolism of chrysene (Seo et al., 2009).

**FIGURE 4:**
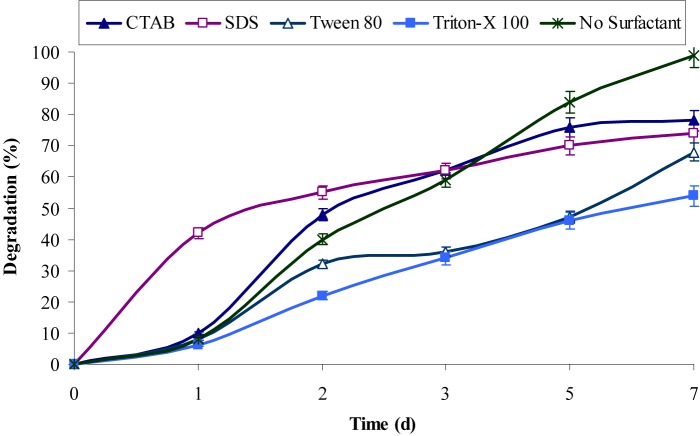
Effect of different surfactants on chrysene degradation by the consortium ASDC under optimized conditions (37°C and pH 7, 150 rpm) in BHM.

The inherent characteristic of many PAHs is to sequester in sediments, which resulted in lower rates of degradation due to their non-availability to bacteria under natural conditions (Boopathy, 2000; Abdel-Shafy and Mansour, 2016). Surfactants molecules were generally found to enhance the metabolism of PAHs bacteria, which either produced by them or supplemented additionally. However, the experimental results from this study revealed that both anionic and cationic surfactants did not enhance chrysene degradation, rather the rate of degradation decreases upon providing extraneous surfactants. **Figure 4** showed that CTAB and SDS decrease chrysene degradation about 1.3-fold (i.e., nearly 76% of 5 mg/L of chrysene was degraded after 7 days). While degradation rate was decreased by 1.45-fold in the presence of Tween-80 and it was also decreased nearly 1.85-fold on supplementing Triton X-100. The decrease in the degradation rate by the consortium in the presence of above surfactants, were either due to their preferential utilization as carbon and energy source ahead of PAHs or toxic to consortial bacteria (Shuttleworth and Cerniglia, 1995; Mrozik et al., 2003; Szczepaniak et al., 2016).

### Effect of Environmental Factors on Chrysene Degradation

Degradation of chrysene always depends on the various operational parameters, which includes physical factors like pH, temperature, oxygen concentration, and chemical factors such as different substrates (PAHs) and/or heavy metals.

#### Effect of Physical Parameters

**FIGURE 5:**
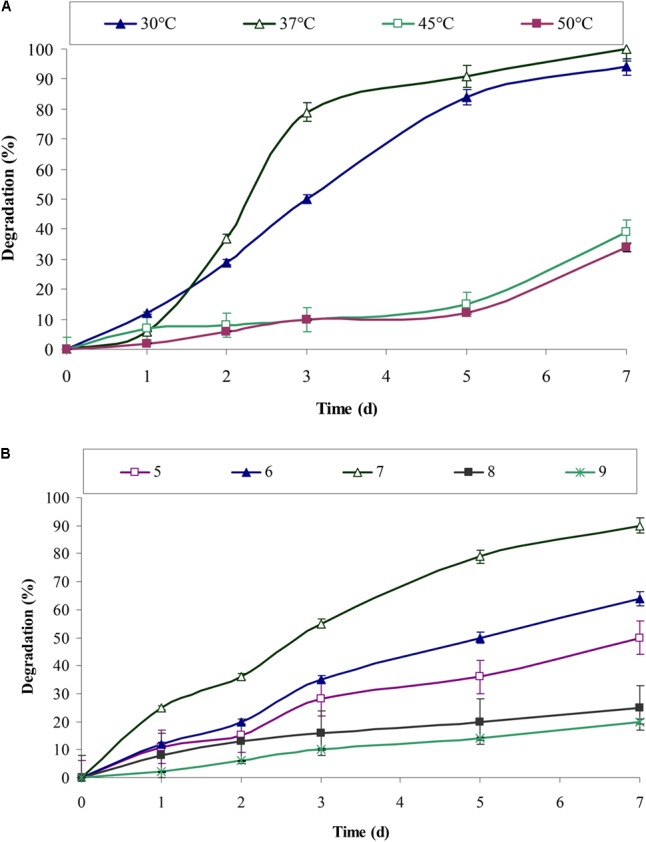
Effect of different **(A)** temperature and **(B)** pH, on the degradation of chrysene by consortium ASDC, grown at 150 rpm in BHM.

pH and temperature are always important parameters and has a significant effect on biochemical and enzymatic reactions. Chrysene degradation by consortium ASDC was pH dependent. **Figure 5B** indicated that efficient degradation was observed toward acidic pH and maximum degradation was obtained at neutral pH. Consortium degraded 64% (of 5 mg/L) of chrysene within 7 days at pH 6, while degradation efficiency reached to 90% at pH 7 and drops sharply with the increase in the pH. The degradation efficiency decreased by 3.6 and nearly 4.5-fold (*P* < 0.05) at pH 9.0 and 10.0 respectively.

In our earlier study on degradation of pyrene by the bacterial consortium ASDP, maximum degradation was observed at pH 7.0 (Vaidya et al., 2017). Various studies have also observed better degradation of different PAHs near neutral pH viz. degradation of octadecane, naphthalene, bioremediation of oily sludge in the soil and gasoline contaminated soil, etc. (Verstraete et al., 1976; Patrick and DeLaune, 1977; Dibble and Bartha, 1979; Hambrick et al., 1980; Leahy and Colwell, 1990). *Rhodococcus* sp. UW1 showed maximum pyrene degradation along with high dioxygenase activity at pH 7.1 and 7.2 respectively (Walter et al., 1991). In artificially contaminated soil, Ravanipour et al. (2015) observed that bacterial consortium showed better degradation at pH 6.8.

The results from **Figure 5A** indicated that chrysene degradation by consortium ASDC was temperature dependent and efficient degradation was observed in the range of 30–37°C. About 94% (of 5 mg/L) of chrysene was degraded at 30°C and efficiency of degradation increases at 37°C, where complete degradation of 5 mg/L of chrysene was observed. On increasing the temperature to 45°C, the degradation rate decreases sharply by 2.6-fold and that for 45°C, it falls by 2.95-fold. Previous studies on degradation of PAHs, observed that most of the aerobic enzymes required for PAHs metabolism have optimum reaction temperatures in the range of 30 to 37°C (Hamzah et al., 2011; Patel et al., 2012a,b; Mukherjee and Roy, 2013; Vaidya et al., 2017). Since the consortium was developed from the soil samples of Amlakhadi canal, which has relatively temperate climatic conditions and temperature ranges between 15 and 45°C (±2°C), the observed results has direct evidence of optimum degradation at 37°C.

Oxygen is a primary requirement for the aerobic metabolism of organic nutrients. Enzymes like monooxygenases and dioxygenases play a significant role in the initial steps of PAHs degradation, which requires molecular oxygen for catalysis (Leahy and Colwell, 1990). The results from this study supports the above notion and it can be observed from Supplementary Figure S1, that under limited concentration of oxygen (i.e., under static condition); rate of degradation of chrysene by consortium was slow, 26% (i.e., only 1.3 mg/L from 5 mg/L) of chrysene was degraded within 7 days. While under same time period, upon increasing the oxygen concentration (by increasing the rpm to 50/100) degradation rate was enhanced between 1.5- and 2.15-fold. But maximum degradation was observed by an increase in 3.5-fold (*P* < 0.05) at 150 rpm, where complete degradation of 5 mg/L of chrysene was observed within 7 days.

#### Effect of Different Substrates (Hydrocarbons and Poly-Aromatic Hydrocarbons)

**FIGURE 6:**
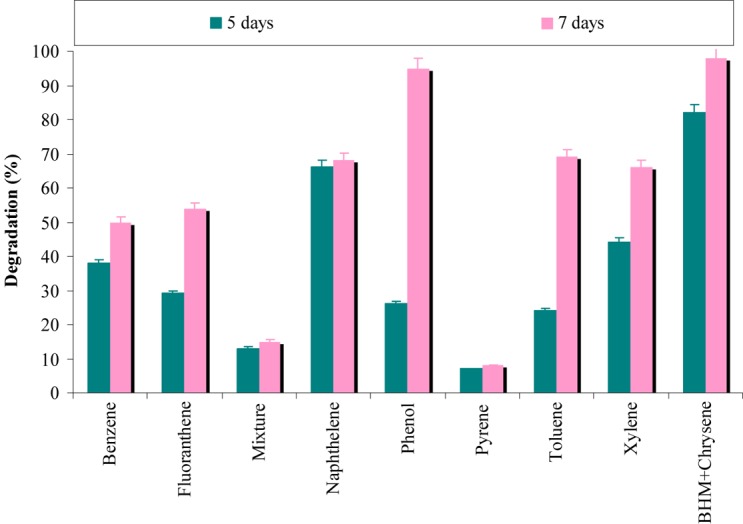
Effect of different aromatic-hydrocarbons on chrysene degradation by the consortium ASDC under optimized conditions of growth (37°C and pH 7, 150 rpm) in BHM.

The results from **Figure 6** revealed that degradation of chrysene was completely inhibited by pyrene, while phenol has a negligible effect on its degradation. In presence of benzene and fluoranthene, more than 50% degradation of chrysene was observed, whereas above 60% degradation was observed in the presence of naphthalene, toluene and xylene. When a mixture of seven PAHs/hydrocarbons (fluoranthene, pyrene, naphthalene, phenanthrene, benzene, toluene, and xylene) was provided; chrysene degradation was decreased by 6.5-fold. The combinatorial effect of a mixture of PAHs/hydrocarbons might have resulted in disruption of the cell membrane of consortial bacteria and have proved to be toxic (Jacques et al., 2005). According to Ascon-Cabrera and Lebeault (1993), bacterial oxygenases have an ability to use more than one hydrocarbon and may co-metabolize different substrates at significantly lower rates.

**FIGURE 7:**
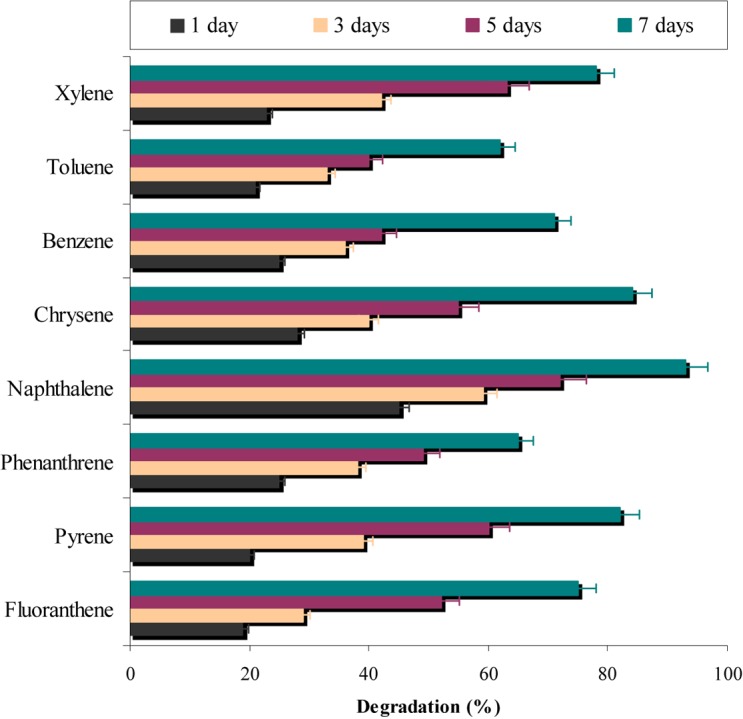
Simultaneous degradation of various aromatic-hydrocarbons along with chrysene degradation by the consortium ASDC under optimized conditions of growth (37°C and pH 7, 150 rpm) in BHM.

In a different set of experiments, results (**Figure 7**) indicated that more than 60% of all the eight PAHs used in the study were degraded by the consortium ASDC. However, the degree of degradation was depended on the presence of PAH and it can be observed that consortium effectively degraded naphthalene (*P* < 0.05), pyrene, fluoranthene, and xylene (≥75%). While ≥62% of phenanthrene, benzene, and toluene were degraded by the consortium under optimized conditions within 7 days. Yuan et al. (2002) observed that metabolic efficiency of microorganism toward pyrene, fluorene, phenanthrene, anthracene, and acenaphthene of was much higher in presence of all five PAHs simultaneously in mixture, when compared to their individual metabolism, since all five substrates either provide more carbon sources or enrichment and acclimatization may enhance degradation rate.

#### Effect of Heavy Metals

**FIGURE 8:**
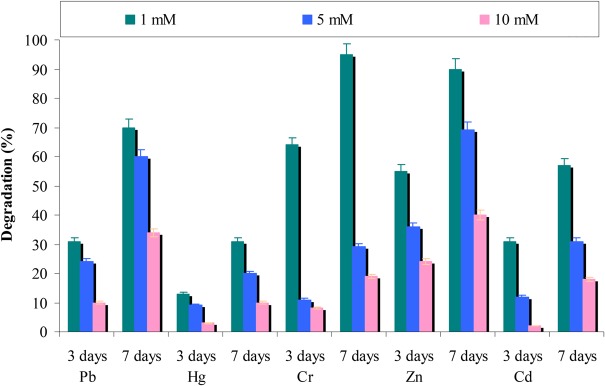
Effect of different heavy metals on chrysene degradation by the consortium ASDC under optimized conditions of growth (37°C and pH 7, 150 rpm) in BHM.

It was found that heavy metals are common pollutants in the environment by industrial activities. Key enzymatic activities in different metabolic reactions essential for microbial growth are inhibited by heavy metals (Chen et al., 2008). Therefore, the effect of heavy metals (in various concentrations) on chrysene degradation was assessed. Results from the **Figure 8** revealed that with an increase in the concentration of heavy metals (from 1 to 10 mM) corresponding decreases in chrysene degradation was observed. The study also suggested that chromium and zinc did not affect chrysene degradation, whereas in presence of lead and cadmium 70 and 57% degradation was observed at 1 mM concentration after 7 days. In the presence of mercury, only 30% chrysene degradation was observed at 1 mM concentration. The results further revealed that consortium ASDC was comparatively resistant toward zinc and lead at their higher concentration than chromium, cadmium, and mercury.

In another experiment, the relationship between growth retardation of consortial species in presence of different concentrations of heavy metals was studied as described by Pepi et al. (2008) in terms of EC_50_ values. From Supplementary Figure S2 it can be observed that EC_50_ Pb^2+^, Hg^2+^, Cr^2+^, Zn^2+^, and Cd^2+^ was 5.6, 4.2, 4.5, 6.0, and 4.4 mM, respectively. Sandrin et al. (2000) suggested that higher tolerance to heavy metals might be due to the production of biosurfactant by the bacterial consortium. The tolerance might also be due to precipitation of heavy metals by phosphate and sulfate of the medium (Hughes and Poole, 1991; Patel et al., 2012b).

### Linear Regression Analysis

The results obtained during the study (i.e., effect of environmental parameters on chrysene degradation) were validated statistically to understand the significance of the observed results. During initial analyzes using ANOVA, factors such as, pH, temperature, oxygen concentration and presence of organic nutrients, found to be significantly (statistically) affecting chrysene degradation. Further analysis was made to find either positive or negative correlations exist between environmental factors and chrysene degradation. Results from **Table 3**, suggested that there exists a strong relationship between temperature, pH, oxygen concentration, organic nutrients and chrysene degradation, since the Pearson’s Correlation Coefficient (i.e., Coefficient of Correlation, *r*) were 0.977, 0.932, 0.978, and 0.871 respectively, indicatively are positively correlated.

**Table 3:** Linear regression correlations for degradation of chrysene by consortium ASDC for parameters found significant by ANOVA analysis.

		Kinetic models		
No.	Factors	Regression equation (Pearson’s regression)	Coefficient of correlation (Pearson’s correlation coefficient) (*r*)	Constant rate
	**pH**			
1	5.0	y = 6.9118x + 2.598	0.989	06.911
2	6.0	y = 9.1765x + 2.637	0.992	09.176
3	7.0	y = 12.706x + 9.382	0.977	12.706
4	8.0	y = 3.2647x + 3.872	0.959	03.264
5	9.0	y = 2.8824x + 0.019	0.994	02.882
	**Temperature (°C)**			
1	30	y = 14.441x + 1.509	0.984	14.441
2	37	y = 15.676x + 5.137	0.932	15.676
3	45	y = 4.8235x − 1.303	0.927	04.823
4	50	y = 4.4118x − 2.568	0.934	04.411
	**Shaking (rpm)/static**			
1	50	y = 5.5x + 2	0.974	05.500
2	100	y = 7.1037x + 8.260	0.953	07.103
3	150	y = 13.055x + 10.024	0.978	13.055
4	Static	y = 3.0366x + 6.682	0.872	03.036
	**Organic nutrient (0.1% w/v)**			
1	Glucose	y = 8.4655x + 14.621	0.871	08.465
2	Phthalic acid	y = 6.0517x + 8.569	0.905	06.051
3	Salicylic acid	y = 10.776x − 0.465	0.983	10.776
4	No nutrient	y = 12.69x + 23.086	0.861	12.690

Therefore, a linear regression analysis was performed to establish the linear correlation between chrysene degradation and pH of the medium, environmental temperature, availability of dissolve oxygen and presence of organic nutrients. Results from **Table 3** suggested that a linear regression was established between all four parameters and chrysene degradation. At 37°C, when *F*(1,4) was 26.882, *P* = 0.006, the effect of temperature was accounted for 83.80% explained variability for chrysene degradation. Similarly for pH 7.0, *F*(1,3) = 84.264, *P* = 0.0007, effect of pH was accounted for 95.468% variability on chrysene degradation, whereas at 150 rpm, *F*(1,3) = 69.53, *P* = 0.003, oxygen concentration had variability of 95.806% for chrysene degradation. Moreover, the half-life for degradation of chrysene at 37°C was 1.23 days, which increased to 4.65 days at 45°C (**Table 4**). Thus, the results for chrysene degradation were statistically analyzed and found to be strongly and linearly correlated with the selected variables for chrysene degradation.

**Table 4:** Half-life values for degradation of chrysene by consortium ASDC under optimized conditions at different temperatures.

No.	Temperature (°C)	t_1/2_ (d)
1	30	3.366
2	37	1.232
3	45	4.651
4	50	2.042

### Degradation Profile of Chrysene and Stoichiometry

**FIGURE 9:**
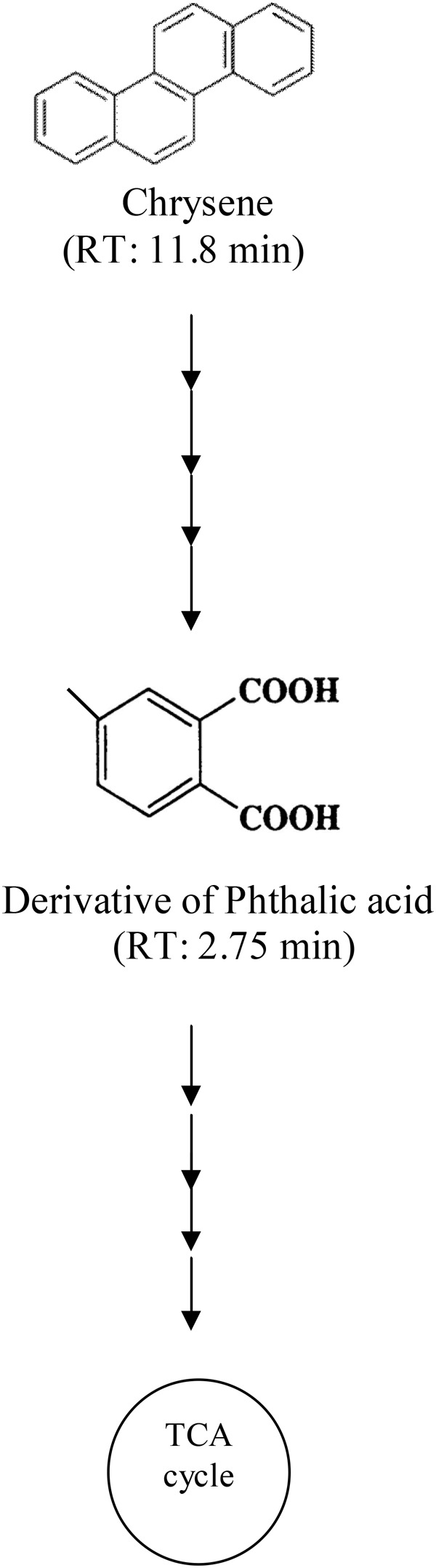
Proposed pathway for chrysene degradation by the consortium ASDC under optimized growth conditions (37°C and pH 7, 150 rpm).

Degradation pattern of chrysene by consortium ASDC was analyzed using HPLC. A single peak of intact chrysene compound was observed at a retention time of 11.8 min in HPLC chromatogram (Supplementary Figure S3). Through the synergistic metabolic activity of consortial bacteria, chrysene was gradually degraded over a period of 7 days, which was evidently observed by a decrease in peak height near 11.8 min from ∼55 mAU (0 days) to 0.6 mAU (7 days) (Supplementary Figures S3a,c). During the degradation process, besides the peak of chrysene, few more peaks were observed on chromatogram and we can postulate the formation of derivative of phthalic acid (a peak near 2.75 min). As a result, it can be proposed that chrysene was degraded through the phthalic acid pathway by the consortium ASDC and the possible pathway of degradation is as depicted in **Figure 9**.

Along with the degradation profile and stochiometric correlation, results from **Table 2** suggested that chrysene degradation by consortium ASDS was non-stoichiometric. At the initial concentration of 21.50 μM (i.e., 5 mg/L) after degradation (i.e., after 7 days) its concentration decreased to 0.90 μM (0.21 mg/L) with simultaneous formation of 1.50 μM (0.31 mg/L) of Phthalic acid. The degradation profile from HPLC chromatogram revealed that there were formations of multiple peaks on the third and 7^th^ day. The results also suggested that a derivative of phenanthrene (phenanthroic acid) might have been formed since a sharp peak at 4.774 min was observed (Supplementary Figure S3b), which overlaps with the retention time of phenanthrene). Nayak et al. (2011) also observed the formation of phenanthroic acid as one of the intermediates during degradation by *Pseudoxanthomonas* sp. PNK-04. Since there was no evidence of accumulation of Phthalic acid, it can be deduced that chrysene was completely mineralized by consortium ASDS. The non-stoichiometric degradation is characteristic of most of the biological processes involving multiple steps.

### Competence of Consortium ASDC for Degradation of Chrysene in Microcosm

The microcosm experiment was designed to access the competence of consortium ASDC for chrysene and PAHs degradation under soil ecosystem in presence of native microflora of polluted and non-polluted soil. Consortium ASDC sharply increases the degradation capability of non-sterile polluted and pristine soils and non-augmented polluted and pristine soils degrade chrysene 73 and 54% respectively whereas augmented sets degrade 96 and 85% respectively in 7 days (**Table 1**). There are very fewer reports of chrysene degradation in microcosms. Nwinyi et al. (2016) observed 14 to 15% degradation of chrysene in 2–3 weeks in laboratory media having a final concentration of 64 mg/kg. But degradation was not examined in microcosm or soil. In this respect, consortium ASDC is quite efficient since, in presence of other compounds, it can degrade chrysene. The abiotic loss is negligible as chrysene is HMW PAH and therefore its tendency to remain in the organic part of the soil. Sterile pristine and polluted soils microcosms augmented by consortium exhibited 64 and 89% degradation which states that components of polluted soil may aid for the degradation as there are presence of many structurally related compounds present in polluted soil which induce the catabolic genes for the catabolism of the aromatic hydrocarbons (Holman et al., 2002; Yang et al., 2011; Li et al., 2013). This is also true for the degradation of chrysene in sterile microcosms of polluted and pristine soils amended by multiple PAHs where 83% chrysene degradation took place in sterile polluted microcosm whereas only 43% degradation occurred in the sterile pristine soil. Non-sterile polluted and pristine soil microcosms amended with multiple PAHs exhibited 67 and 60% degradation of chrysene. This may be due to the concentration effect of other PAHs which are present in higher concentrations and so degraded first than chrysene which is present in 5 mg/kg concentration whereas other PAHs are present in 100, 250, and 500 mg/kg concentrations.

## Conclusion

In this study, the effective competence of an enriched consortium was described for its inherent potential to metabolize and degrade chrysene as the sole source of carbon and energy through phthalic acid degradation pathway. It further revealed that, dissolved oxygen concentration, pH and temperature have profound effect on chrysene degradation, while glucose and pyrene inhibited chrysene degradation. The above study also suggested that the degradation ability of developed consortium can be further used in higher macrocosm or reactor scale studies. Because results (in microcosms study) revealed that with the low nutrient requirement, the growth of the consortium ASDC and the degradation potential of the chrysene were largely unaffected even in the presence of other PAHs and heavy metals.

Therefore consortium ASDC is efficient in degradation of chrysene in presence of different PAHs and aromatic hydrocarbons, degradation of multiple hydrocarbons and PAHs and efficient degradation in microcosms of chrysene and other pollutant compounds.

## Author Contributions

SV performed the experiments and contributed in manuscript writing. ND manuscript design and contributed in manuscript writing. KJ experimental design and manuscript writing. DM provided concept and manuscript writing.

## Conflict of Interest Statement

The authors declare that the research was conducted in the absence of any commercial or financial relationships that could be construed as a potential conflict of interest.
