# Supplementary material for: Degradation of Chrysene by Enriched Bacterial Consortium
Source: Front Microbiol. 2018 Jun 26;9:1333. doi: 10.3389/fmicb.2018.01333 (PMC6036299; doi:10.3389/fmicb.2018.01333)
Supplement: Supplementary file 1 [file Data_Sheet_1.pdf]

Supplementary Information

Degradation of chrysene by enriched bacterial consortium

Sagar Vaidya[#], Neelam Devpura[#], Kunal Jain, Datta Madamwar

Environmental Genomics and Proteomics Lab, UGC Centre of Advanced Study, P.G. Department of Biosciences, Satellite Campus, Vadtal Road, Sardar Patel University, Bakrol – 388 315, Anand, Gujarat, India

Shares Equal Authorship

*** Corresponding author**

Tel: +91 9825686025

Fax: +91 2692 236475

Email addresses: sagarvaidya207@gmail.com (S. Vaidya)

ndneelam13@gmail.com (N. Devpura)

datta_madamwar@yahoo.com (D. Madamwar)

Table S1: Degradation of chrysene by various bacteria/fungi

No.	Organisms	Initial chrysene concentration (mg/L)	Degradation efficiency (%)	Time required (days)	Co-substrates	Reference
1	*Rhizomonas* & *Sphingomonas*	500	97	35	Mineral oil	Willison, 2004
2	*Polyporus* sp.	228	58	30	Polypeptone, Tween 20, Tween 80	Hadibarata et al., 2009
3	*Pseudoxanthomonas* sp.	400	60	12	---	Nayak et al., 2011
4	*Fusarium* sp.	228	48	30	Polypeptone, Tween 80	Hidayat et al., 2012
5	*Alkaligenes faecalis*	10-100	Resistant to 90 mg/l and exhibited growth when exposed to 10 mg/l	5	---	John et al., 2012
6	*Bacillus* sp. & *Pseudomonas* sp.	50	15 & 17	7	---	Monika et al., 2010
7	*Mycobacterium parmense*	20	62	30	---	Salvador et al., 2009
8	*Pichia anomala*	25	7.6	10	Tween 80	Abd El-Latif et al., 2006
9	*Paracoccus* sp.	20	100	16	In presence of other PAHs like nap.., phe.., anth.. and fluo….	Haimau et al., 2004
10	Bacterial consortium of *Rhodococcus* sp., ASDC1; *Bacillus* sp. ASDC2 and *Burkholderia* sp. ASDC3	1-10	100	7	As sole source of carbon and energy	This Study

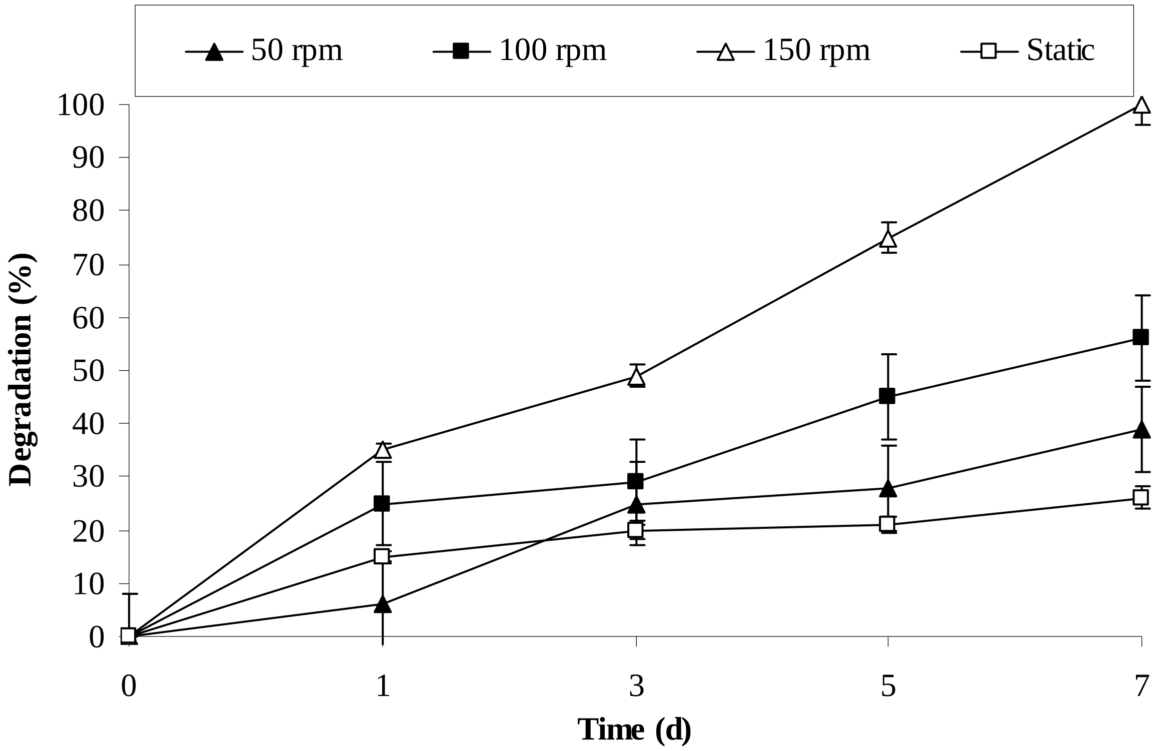

Figure S1: Effect of shaking (speed) and static conditions on the degradation of chrysene by consortium ASDC grown under optimized conditions (37ºC and pH 7, 150 rpm)

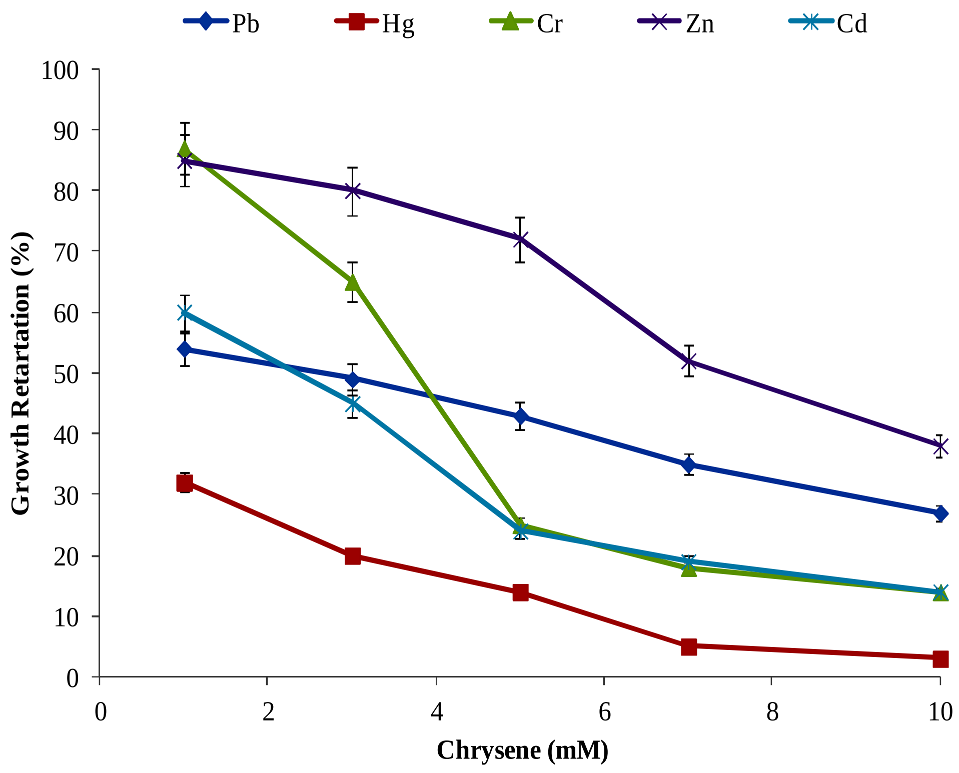

Figure S2: The effect of heavy metals on percent growth retardation of the consortium ASDC, during degradation of chrysene

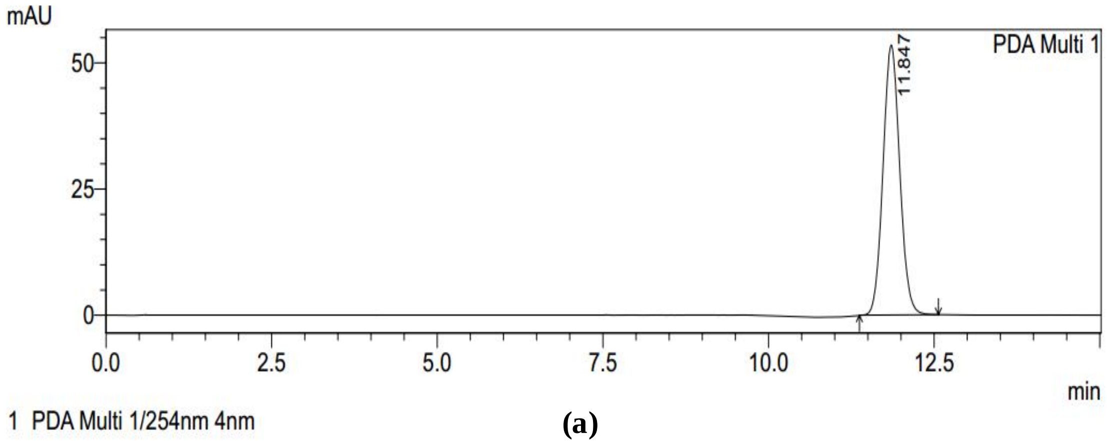

(a)

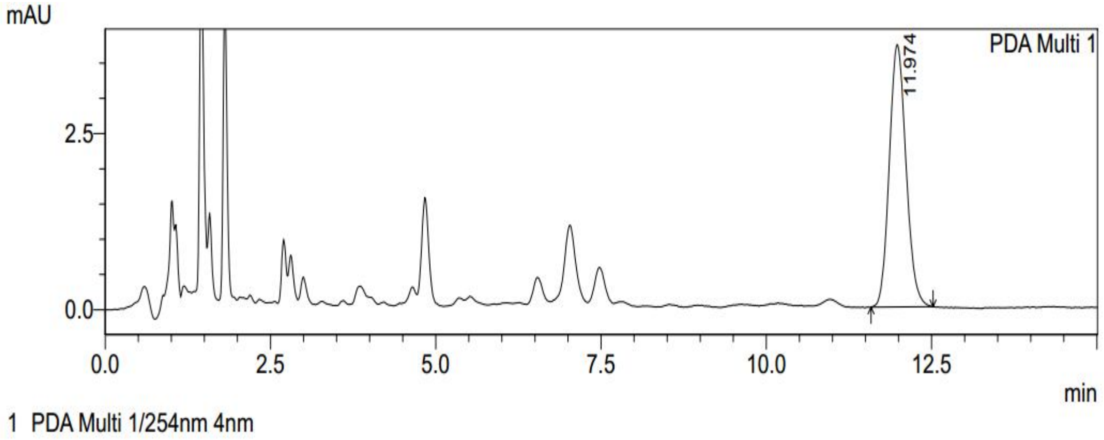

(b)

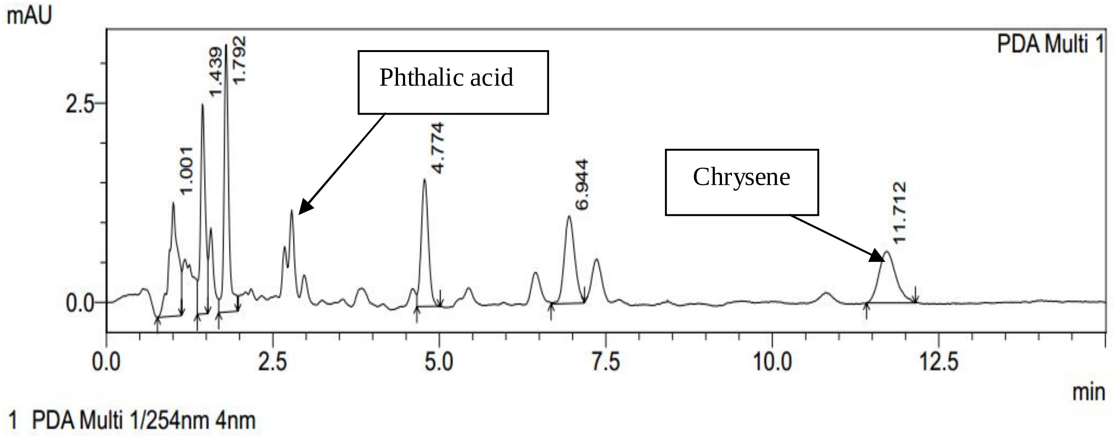

(c)

Figure S3: HPLC chromatogram of (a) intact chrysene, (b) degraded intermediates after 3 d and (c) degraded intermediates after 7 d
